# Acute Lymphoblastic Leukemia in a Patient With Advanced Breast Cancer Treated With Cyclin-Dependent Kinase 4/6 Inhibitors and Endocrine Therapy

**DOI:** 10.7759/cureus.69548

**Published:** 2024-09-16

**Authors:** Ryan E Bailey, Marcela Mazo Canola

**Affiliations:** 1 School of Medicine, Long School of Medicine, San Antonio, USA; 2 Breast Medical Oncology, MD Anderson Cancer Center, San Antonio, USA

**Keywords:** b-cell acute lymphoblastic leukemia, acute lymphoblastic leukemia, aromatase inhibitor therapy, cdk 4/6 inhibitors, breast cancer management

## Abstract

This case shares the case of a post-menopausal woman who develops Philadelphia chromosome-positive B cell acute lymphoblastic leukemia (B-ALL) while receiving treatment for invasive ductal carcinoma (IDC) of the breast. The patient received a cyclin-dependent kinase (CDK) 4/6 inhibitor + aromatase inhibitor (AI) for the IDC; hyperfractionate cyclophosphamide, vincristine sulfate, doxorubicin hydrochloride (Adriamycin), methotrexate, and cytarabine (hyperCVAD), and the steroid hormone dexamethasone were added to treat the B-ALL. HyperCVAD combined with CDK 4/6 inhibitor + AI was very well tolerated. The CDK 4/6 inhibitor and AI were only held once in the treatment course due to adverse effect (AE) intolerance. The patient remains on a CDK 4/6 inhibitor and ponatinib with only low-grade fatigue as an AE. This case underscores the importance of a concurrent approach to managing hematologic and breast malignancies. The combined treatment regimens were effective and well-tolerated. Vigilant follow-up is essential for patients in remission from both malignancies, ensuring effective disease surveillance and treatment management. Integrated care remains pivotal for optimal outcomes.

## Introduction

Breast cancer is a common malignancy in women in the United States (30% of female cancer diagnoses) and worldwide (25% of female cancer diagnoses) [1]. Diagnosis is uncommon in women younger than 40 but incidence increases progressively peaking with women in their 70s [1]. Infiltrating ductal carcinoma is the most common type of invasive breast cancer, composing 70-80% of invasive neoplasms [2].

PALOMA-2, a randomized clinical trial initiated in 2013, compared cyclin-dependent kinase (CDK) 4/6 inhibitors (palbociclib) plus aromatase inhibitors (AIs) (letrozole) versus AIs as a single agent [3]. Palbociclib, a CDK 4/6 inhibitor, was approved by the Food and Drug Administration (FDA) in February 2015 for the treatment of estrogen receptor positive (ER+) and human epidermal growth factor receptor 2 (HER2)-negative advanced breast cancer as initial endocrine-based therapy in postmenopausal women. CDK 4/6 inhibitors combined with AIs have been shown to improve progression-free survival (PFS), objective response rate (ORR), and clinical benefit rate (CBR) compared with AI monotherapy [4]. Palbociclib blocks cell cycle advancement from G1 to S phase. The side effect profile of palbociclib aligns with many other chemotherapeutic agents. Common adverse effects (AEs) include neutropenia (rarely neutropenic fever), leukopenia, lymphopenia, nausea, infections, fatigue, and diarrhea [5].

Acute lymphoblastic leukemias (ALLs) and lymphomas are hematologic malignancies that arise from cells of the B or T cell lineage in the bone marrow and lymphatic tissues, respectively. The estimated incidence of ALL in the United States is 1.6 cases/100,000 people [6]. Greater than 66% of all ALLs are B cell ALLs (B-ALL) [7,8]. B-ALL is much more common in children (75% of cases occur in children <6 years of age (YOA)), but a second peak in incidence occurs in late adulthood (~60 YOA) [7,8]. B-ALL has different presentations; some patients’ disease takes an insidious time course while others experience acute disease. Many patients with B-ALL present with history of anemia, neutropenia, and/or thrombocytopenia. Fatigue, easy bleeding, frequent infections, and dysarthria are common also. Systemic symptoms of B-ALL are fever and weight loss, although these are frequently mild [9]. B-ALL can be differentiated from T cell ALL (T-ALL) and other malignancies by immunohistochemistry and flow cytometry. CD19, CD79a, CD22 (B cell), CD10, CD24, PAX5, and TdT (lymphoblast) positivity and CD3 (T cell) and MPO (myeloid) negativity strongly support a B-ALL diagnosis [10].

Many subtypes exist for acute leukemia beyond the T/B cell distinction. Philadelphia positive (Ph+; t(9;22)(q34.1; q11.2); BCR-ABL1 subtype, referred to as Ph+ B-ALL) has been seen as a negative prognostic indicator; however, with the advent of Imatinib (Gleevec), it is unclear if this is still the case [11]. The clinical presentation of Ph+ B-ALL is generally the same as Ph- B-ALL. Ph+ and its treatment consist of allogeneic hematopoietic cell transplant (allo-HCT), tyrosine kinase inhibitors (TKIs), and hyper-CVAD combination chemotherapy [12,13].

This article presents the case of a woman diagnosed with Ph+ B-ALL while undergoing letrozole and palbociclib treatment for metastatic, HR+, HER2/neu-, advanced invasive ductal carcinoma (IDC).

## Case presentation

A woman in her 40s presented to one of our affiliate institutions in 2013 with the chief complaint of progressively worsening neck pain and shortness of breath (SOB) when lying on her left side. She had no significant past medical history and was not taking medications. CT was done as part of her workup (images from outside institution and unavailable), showing left pleural effusion with partial collapse of the lower left lobe (LLL) and a 4-mm right middle lobe (RML) nodule as well four distinct masses in the right breast: two spiculated masses at the 2 and 3 o’clock position and two smaller masses located anteriorly and posteriorly. At least one of the masses appeared to invade the pectoralis muscle with associated axillary lymphadenopathy.

An ultrasound of her right breast (images from outside institution and unavailable) revealed two abnormal hypoechoic irregular masses at 2 o’clock measuring: 1.9 cm x 2.1 cm x 1.6 cm and a more superior mass 2.4 cm x 1.5 cm x 1.7 cm. enlarged axillary lymph nodes were seen and measured at 2.6 x cm 1.5 cm x 1.4 cm.

**Figure 1:**
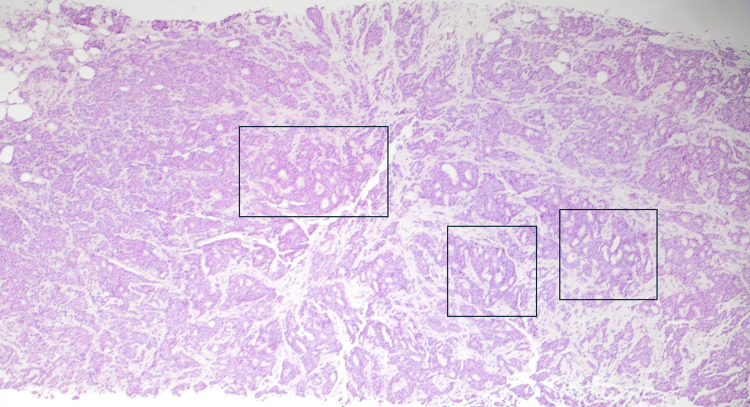
Arches of neoplastic cells in a cribriform pattern accompanied by desmoplastic stroma (hematoxylin and eosin, 40x magnification) Black boxes outline areas of cribriform architecture.

**Figure 2:**
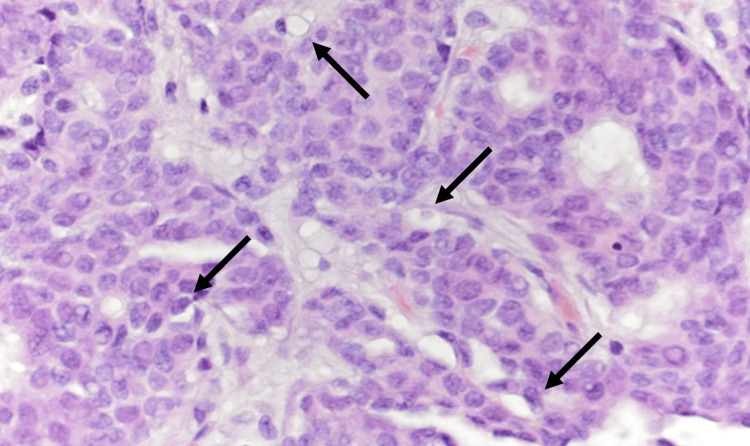
Neoplastic cells display nuclei with vesicular chromatin and mild pleomorphism (hematoxylin and eosin, 400x magnification) Arrows denote cells with vesicular chromatin.

**Figure 3:**
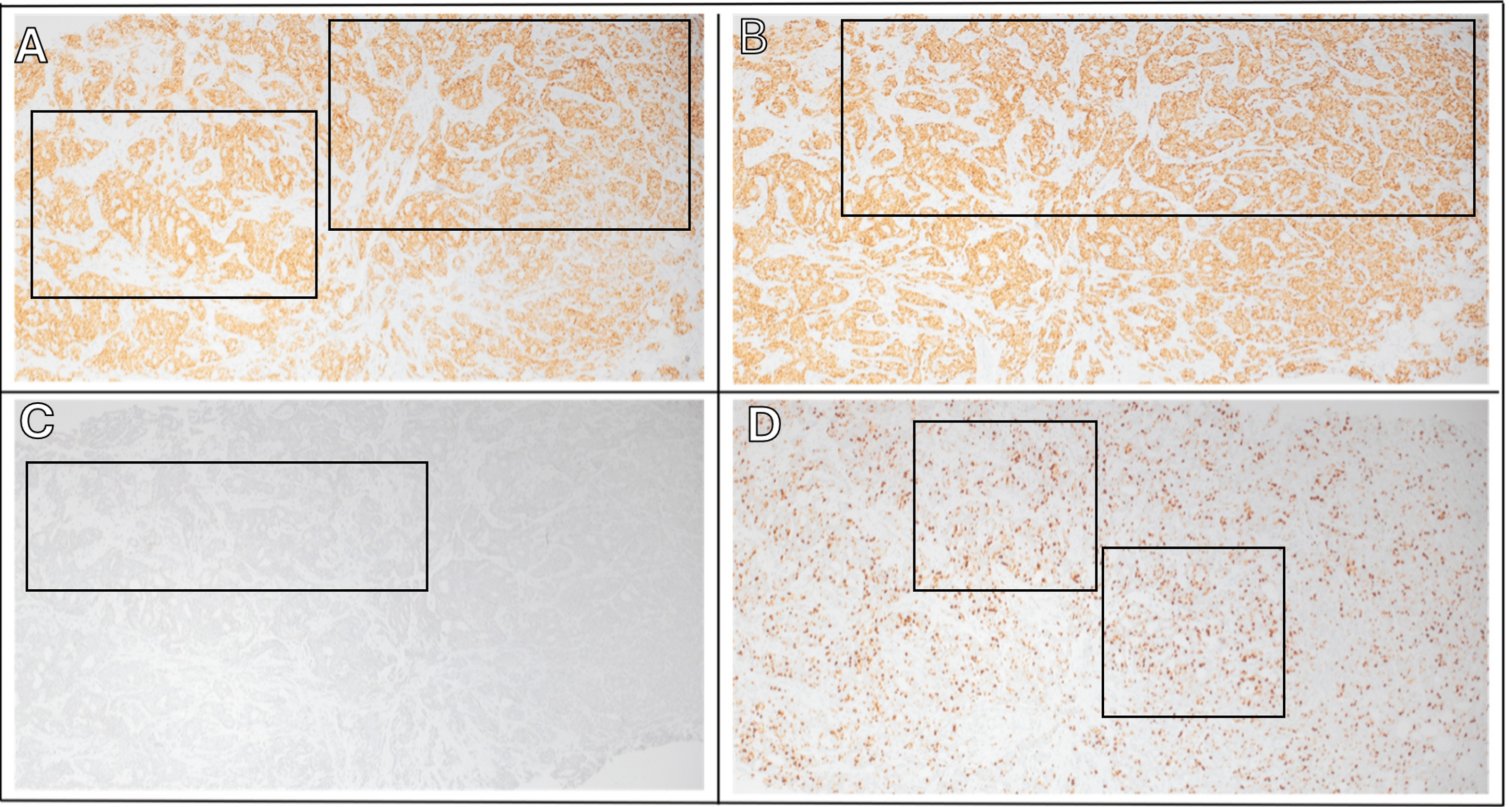
Immunohistochemical staining of breast biopsy (A) >95% ER positivity with strong intensity, 10x magnification; (B) >95% ER positivity with strong intensity, 40x magnification; (C) >95% PR positivity with strong intensity, 10x magnification; (D) >95% PR positivity with strong intensity, 40x magnification. Regions of enhanced signal intensity have been demarcated by black outlines. ER: Estrogen receptor; PR: Progesterone receptor

Sonogram-guided core needle biopsy in one of the breast masses revealed an IDC with adenoid cystic features. Figures 1 and 2 depict the cribriform architecture and pleomorphism of the cells in the biopsy. The prognostic factor panel showed estrogen receptor (ER) positivity of 62.5% (Figure 3A), progesterone receptor (PR) positivity of 62.5% (Figure 3B), HER2 positivity of 0% (not shown), and Ki-67 positivity of 50% (not shown). 

The patient was started on goserelin for ovarian function suppression and tamoxifen one month later. The patient responded well to tamoxifen; tumor mass and axillary LAD decreased to the point that they could not be clearly palpated. The patient experienced several AEs of tamoxifen, including hot flashes, vaginal pruritis, and decreased sex drive. Tumor markers decreased as well. The patient received an oophorectomy the following year. In 2015, the patient was declared to have stable disease, but six months later, her tumor markers rose again, which was a finding corroborated by breast exam. Letrozole and palbociclib were added to her treatment regimen. By 2018, she was declared to be in complete clinical remission after three years without evidence of recurrent disease. The patient continued current therapy with CDK 4/6 inhibitor and AI.

**Figure 4:**
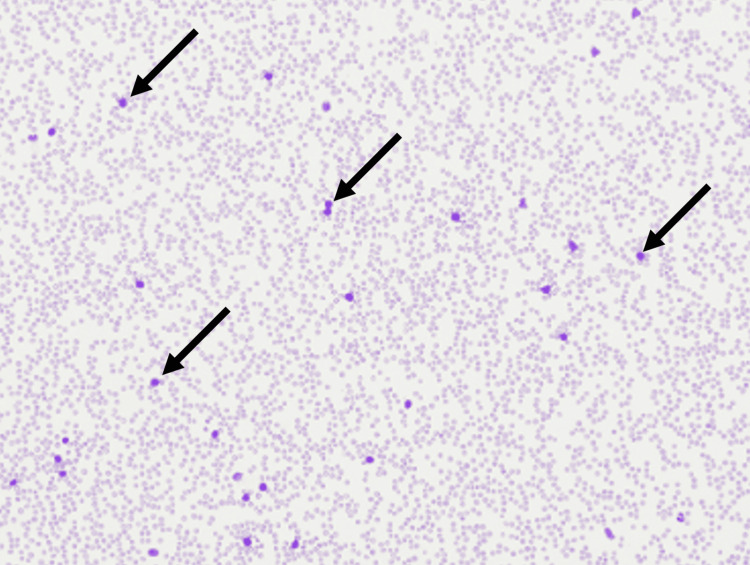
Low power peripheral blood smear showing blasts Black arrows indicate blasts.

**Figure 5:**
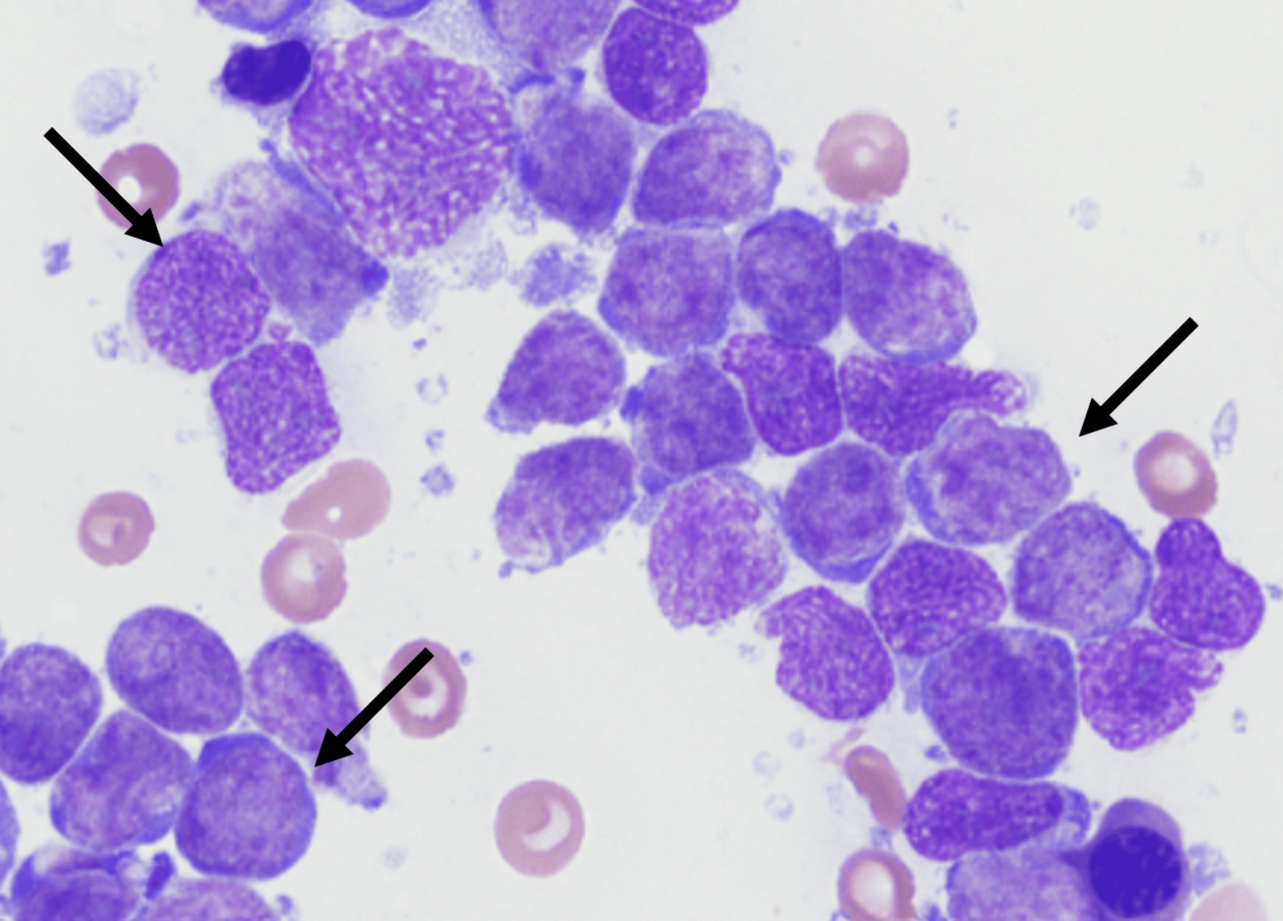
Blasts in the bone marrow touch preparation Arrows indicate some of the blasts in the figure.

**Figure 6:**
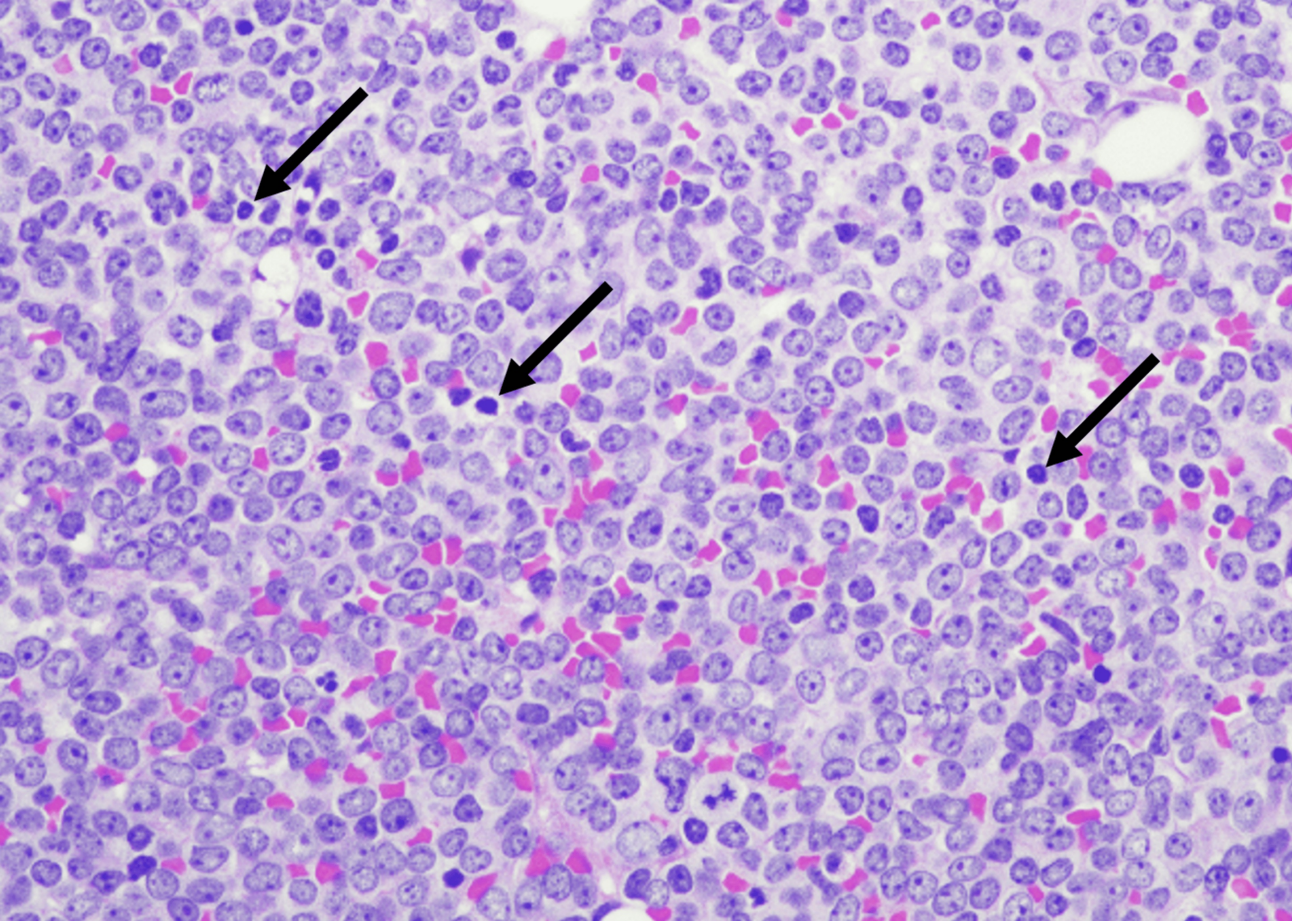
Bone marrow core biopsy, high power Arrows indicate blasts in the bone marrow.

The patient completed hyperfractionate cyclophosphamide, vincristine sulfate, doxorubicin hydrochloride (Adriamycin), methotrexate, and cytarabine (hyperCVAD) three months later and continues to take dasatinib daily. A repeated bone marrow biopsy seen in Figures 4, 5, 6, showed a complete response with no evidence of molecular residual disease. The patient continues to take letrozole as a single agent. Palbociclib was discontinued after the B-ALL diagnosis. The last set of CT and bone scans confirm that the patient is in remission from breast cancer.

In 2021, the patient presented to our institution complaining of mild fatigue, rash, headache, and insomnia. She was still adherent to maintenance letrozole and palbociclib at the time. Complete blood count (CBC) depicted in Table 1, revealed Grade 4 thrombocytopenia , leukocytosis, and megaloblastic anemia. Palbociclib was held for one week, and CBC was repeated. The second CBC showed thrombocytopenia and leukocytosis to be more severe than before, and the patient was admitted into the inpatient service. An urgent bone marrow biopsy was done due to persistent cytopenia. Bone marrow analysis revealed 90% percent blasts. Flow cytometry revealed cells with low CD45 positivity and CD19, Cd22, CD10, CD34, TDT positive, and CD20 negative cells, confirming the diagnosis of ALL. Her disease was later classified as Ph+ (unfavorable prognosis) via chromosomal analysis. Between 2021 and 2023, the patient received four cycles of hyperCVAD and dasatinib.

Outcome and follow-up

The patient experienced a blast crisis two years later, requiring inpatient admission. A lumbar puncture reveals CNS positive for blasts. The patient was started on Blinatumomab with Ponatinib. A restaging bone marrow biopsy after cycle 1 showed complete response with no evidence of molecular residual disease. The patient remains on Blinatumomab + Ponatinib. She experiences few AEs other than fatigue. The fatigue does not affect her activities of daily living (ADLs) or quality of life (QOL). She will continue monthly outpatient monitoring.

**Table 1:** CBC at presentation in 2021 MCV: Mean corpuscular volume; CBC: Complete blood count

Parameters	Value	Reference Range
White Blood Cell Count	20.7 x 10^3^ / µL	4.8 - 10.8 x 10^3 ^/ µL
Hemoglobin	11.5 g /dL	12.0 - 16.0 g /dL
Hematocrit	32.6 %	37.0 - 47.0 %
MCV	104.5 fL	81.0 - 99.0 fL
Platelet Count	22 x 10^3^ / µL	130 - 400 x 10^3^ / µL

## Discussion

CDK 4/6 inhibitors

CDK 4/6 inhibitors exert their effects on neoplastic cells via various mechanisms. The most well-documented mechanism involves the induction of cytostasis. CDK 4/6 inhibitors bind to the adenosine triphosphate (ATP)-binding domain of CDKs 4 and 6, causing cell cycle arrest at the G1-S checkpoint. This effect is most pronounced in retinoblastoma (RB) proficient cells. Another mechanism by which CDK 4/6 inhibitors suppress cancer cells is via epigenetic remodeling. They reprogram the active enhancer landscape in an RB-dependent manner. The enhancers activated by CDK 4/6 inhibitors control processes, including luminal differentiation, resistance to apoptosis, and tumor cell immunogenicity. Interestingly, CDK 4/6 inhibitors do not seem to kill neoplastic cells directly. This is supported by growing evidence that these drugs promote evasion of apoptosis and autophagy, which is consistent with their pro-senescent primary mechanism. Both mechanisms are somewhat disputed but illustrate the complex role that CDK 4/6 inhibitors hold in anti-neoplastic drug regimens. Finally, CDK 4/6 inhibitors promote an inflammatory tumor microenvironment, leading to the activation of effector T cells. The increased immunogenicity induced by CDK 4/6 inhibitors contributes to these agents’ anti-tumor activity and is the prominent means by which they indirectly cause tumor cell death [14]. Food and Drug Administration (FDA) approved palbociclib in 2015 for advanced breast cancer and thus has become a powerful therapeutic tool for advanced, ER+ HER2- breast cancer treatment. It is standard of care to use CDK 4/6 inhibitors in conjunction with an AI.

Palbociclib has not been demonstrated to have long-term cumulative or delayed toxicities. Cytopenias are common AEs associated with these agents. Of these, neutropenia is the most frequently reported; despite this, febrile neutropenia is quite rare among the treatment group. Neutropenia grade remained relatively stable throughout the assessment period indicating that Palbociclib’s toxicity to bone marrow was not cumulative. Other hematologic toxicities include cytopenias not limited to leukopenia, thrombocytopenia, anemia, and lymphopenia. Non-hematologic AEs include alopecia, hot flashes, fatigue, nausea, vomiting, diarrhea, pyrexia, stomatitis, and arthralgia [15].

Prognostication

Once the diagnosis of B-ALL has been established, there are several indicators to estimate disease prognosis. Age serves as a major prognostic factor in which older children tend to have better outcomes than young children and adults. Being Black and/or Hispanic is associated with adverse outcomes compared to non-Black and/or non-Hispanic. Leukocyte count > 50,000 / µL and the male sex are both associated with poorer outcomes. Many molecular phenotypes exist, such as hyperdiploid ALLs and ETV-RUNX1 mutant ALL being relatively low risk. Crucial to our case is the Philadelphia chromosome-positive ALL with a five-year survival rate less than 70%, placing this subtype among other high-risk ALL subtypes [16-18].

Treating ALL

Treatment of ALL is broken into three phases: induction, consolidation/intensification, and maintenance. Induction therapy is designed to destroy most of the blasts in the peripheral blood and bone marrow. There are many drug regimens and combination therapies for treating ALL; because there have been few trials comparing induction therapies to each other, few recommendations for induction are set. In this case, the patient received hyperCVAD as induction therapy, but other potential options include French Group of Research on Adult ALL (GRAALL) 2003 or GRAALL 2005, or the Cancer and Leukemia Group B (CALGB) ALL regimen. These regimens are often intensified or modified during the consolidation treatment phase and deintensified during maintenance. Several trials have considered adding rituximab during the maintenance stage.

Philadelphia chromosome-positive ALL

There are special treatment considerations for Philadelphia chromosome-positive ALL. Because oncogenesis is being driven at least partly by the BCR-ABL chimera (the result of the Philadelphia chromosome), an anti-BCR-ABL TKI such as dasatinib must be added to the induction regimen. The anti-BCR-ABL TKI must be paired with either a glucocorticoid or chemotherapy. Choosing between a glucocorticoid and chemotherapy should depend largely on the patient’s age, comorbidities, and functional status. Evidence supports chemotherapy as more effective at achieving a robust molecular response; however, this comes at the cost of higher morbidity. Glucocorticoid + TKI still achieves high rates of complete remission with much less morbidity and mortality.

Treating double cancers

There are many treatment considerations for treating patients with two or more distinct malignancies. Medication interactions, AE tolerance, and insurance coverage are all relevant to determining treatment goals and therapeutic selection. Even the language used to measure treatment effect, overall survival, PFS, and no evidence of disease are all geared towards patients with singular disease or metastatic disease with a single primary site. These considerations have been well-documented for esophageal with concomitant solid malignancy [19]. In some countries, getting insurance coverage for therapeutics may be difficult if certain anti-cancer therapeutics are not indicated treatment of one of a patient's multiple cancers. Treatment considerations for 'double cancers' remain highly individualized. Much more research is needed to build out true clinical algorithms for patients with multiple distinct malignancies.

## Conclusions

Concomitant hematologic and breast malignancies present a unique challenge in selecting a treatment regimen. Concerns regarding medication interactions and a patient’s ability to tolerate two chemotherapeutic regimens are front of mind. This case illustrates that the oncologist need not compromise. The appropriate regimen for IDC can be continued while initiating hyperCVAD for B-ALL. We believe that this patient’s Ph+ B-ALL arose independently of the patient’s history of breast cancer and CDK 4/6 inhibitor + AI treatment. Treatments for hematologic malignancies can sometimes increase the risk of future cancer. There is no evidence that the inverse of this relationship exists in oncology. Rigorous and consistent follow-up becomes all the more important in patients with multiple malignancies. This is integral not only for continued disease surveillance but also for the monitoring of potential drug-drug interactions and toxicities.

## References

[1] Houghton SC, Hankinson SE (2021). Cancer progress and priorities: breast cancer. Cancer Epidemiol Biomarkers Prev.

[2] Memon G, Alkabban FM, Ferguson T (2024). Breast cancer. https://www.ncbi.nlm.nih.gov/books/NBK482286/.

[3] Finn RS, Martin M, Rugo HS (2016). Palbociclib and letrozole in advanced breast cancer. N Engl J Med.

[4] Shimoi T, Sagara Y, Hara F, Toyama T, Iwata H (2020). First-line endocrine therapy for postmenopausal patients with hormone receptor-positive, HER2-negative metastatic breast cancer: a systematic review and meta-analysis. Breast Cancer.

[5] Spring LM, Zangardi ML, Moy B, Bardia A (2017). Clinical management of potential toxicities and drug interactions related to cyclin-dependent kinase 4/6 inhibitors in breast cancer: practical considerations and recommendations. Oncologist.

[6] Terwilliger T, Abdul-Hay M (2017). Acute lymphoblastic leukemia: a comprehensive review and 2017 update. Blood Cancer J.

[7] Dores GM, Devesa SS, Curtis RE, Linet MS, Morton LM (2012). Acute leukemia incidence and patient survival among children and adults in the United States, 2001-2007. Blood.

[8] Redaelli A, Laskin BL, Stephens JM, Botteman MF, Pashos CL (2005). A systematic literature review of the clinical and epidemiological burden of acute lymphoblastic leukaemia (ALL). Eur J Cancer Care (Engl).

[9] Ducassou S, Ferlay C, Bergeron C (2011). Clinical presentation, evolution, and prognosis of precursor B-cell lymphoblastic lymphoma in trials LMT96, EORTC 58881, and EORTC 58951. Br J Haematol.

[10] (2023). WHO classification of tumours of haematopoietic and lymphoid tissues. https://www.iarc.who.int/news-events/who-classification-of-tumours-of-haematopoietic-and-lymphoid-tissues-2/.

[11] Schultz KR, Devidas M, Bowman WP (2014). Philadelphia chromosome-negative very high-risk acute lymphoblastic leukemia in children and adolescents: results from Children's Oncology Group Study AALL0031. Leukemia.

[12] Ravandi F (2019). How I treat Philadelphia chromosome-positive acute lymphoblastic leukemia. Blood.

[13] Kantarjian HM, O'Brien S, Smith TL (2000). Results of treatment with hyper-CVAD, a dose-intensive regimen, in adult acute lymphocytic leukemia. J Clin Oncol.

[14] Watt AC, Goel S (2022). Cellular mechanisms underlying response and resistance to CDK4/6 inhibitors in the treatment of hormone receptor-positive breast cancer. Breast Cancer Res.

[15] Diéras V, Rugo HS, Schnell P (2019). Long-term pooled safety analysis of palbociclib in combination with endocrine therapy for HR+/HER2- advanced breast cancer. J Natl Cancer Inst.

[16] Dunsmore KP, Devidas M, Linda SB (2012). Pilot study of nelarabine in combination with intensive chemotherapy in high-risk T-cell acute lymphoblastic leukemia: a report from the Children's Oncology Group. J Clin Oncol.

[17] Asselin BL, Devidas M, Wang C (2011). Effectiveness of high-dose methotrexate in T-cell lymphoblastic leukemia and advanced-stage lymphoblastic lymphoma: a randomized study by the Children's Oncology Group (POG 9404). Blood.

[18] Schultz KR, Carroll A, Heerema NA (2014). Long-term follow-up of imatinib in pediatric Philadelphia chromosome-positive acute lymphoblastic leukemia: Children's Oncology Group study AALL0031. Leukemia.

[19] Sohda M, Yokobori T, Kimura A (2023). Efficacy of chemotherapy for comorbid cancer in patients with simultaneous double cancers: a multicenter study. Surg Today.

